# Validity and clinical utility of the obsessive compulsive inventory - child version: further evaluation in clinical samples

**DOI:** 10.1186/s12888-020-2450-7

**Published:** 2020-02-03

**Authors:** Kristina Aspvall, Matti Cervin, Per Andrén, Sean Perrin, David Mataix-Cols, Erik Andersson

**Affiliations:** 1 Centre for Psychiatry Research, Department of Clinical Neuroscience, Karolinska Institutet & Stockholm Health Care Services, Region Stockholm, Stockholm, Sweden; 2 Faculty of Medicine, Department of Clinical Sciences Lund, Lund University & Skåne Child and Adolescent Psychiatry, Lund, Sweden; 3 Department of Psychology, Lund University, Lund, Sweden

**Keywords:** Obsessive-compulsive disorder, Pediatric, Child, Adolescent, Psychometric, Inventory, Self-report, Symptoms

## Abstract

**Background:**

Obsessive-compulsive disorder (OCD) is a clinically heterogeneous disorder. Currently, the Obsessive Compulsive Inventory-Child Version (OCI-CV) is the only self-report measure that fully captures this symptom heterogeneity in children and adolescents. The psychometric properties of the OCI-CV are promising but evaluations in large clinical samples are few. Further, no studies have examined whether the measure is valid in both younger and older children with OCD and whether scores on the measure are elevated in youths with OCD compared to youths with other mental disorders.

**Methods:**

To address these gaps in the literature, we investigated the psychometric properties and validity of a Swedish version of the OCI-CV in a large clinical sample of youth aged 6–18 years with OCD (*n* = 434), anxiety disorders (*n* = 84), and chronic tic disorders (*n* = 45).

**Results:**

Internal consistency coefficients at the total scale and subscale level were consistent with the English original and in the acceptable range. Confirmatory factor analyses revealed an adequate fit for the original six-factor structure in both younger and older children with OCD. Correlations between total scores on the OCI-CV and the Children’s Yale-Brown Obsessive-Compulsive Scale (CY-BOCS) were small at pre-treatment (*r* = 0.19) but large at post-treatment (*r* = 0.62). Youth with OCD scored higher than those with anxiety and chronic tic disorders, and the OCI-CV was sensitive to symptom change for youth undergoing treatment for OCD.

**Conclusions:**

This Swedish version of the OCI-CV appears to be a valid and reliable measure of the OCD symptom dimensions across age groups and has good clinical utility.

## Introduction

The international standard for the assessment of symptom severity and outcome in treatment trials of pediatric obsessive-compulsive disorder (OCD) is the Children’s Yale-Brown Obsessive-Compulsive Scale (CY-BOCS [1, 2]). The CY-BOCS is a clinician-administered interview that includes a symptom checklist followed by severity ratings for obsessions and compulsions, yielding an overall severity score. Although the CY-BOCS is a valid and highly sensitive measure of OCD severity, self-report scales have certain advantages over interview-based approaches [3]. For instance, self-report scales with sustained precision and validity have the potential to save time and resources for clinicians, which in turn can make assessments of OCD more scalable and facilitate cost-effective ways of evaluating interventions. According to a review by Rapp et al. [2], there are several self-reported measures for pediatric OCD: the CY-BOCS-Child Report [4]; the Obsessive Compulsive Inventory – Child Version (OCI-CV [5]); the Children’s Florida Obsessive Compulsive Inventory (C-FOCI [6]); and the Children’s Obsessional Compulsive Inventory (ChOCI [7]). Although all of these are promising measures to assess OCD symptoms, the OCI-CV [5] is the only self-report measure that captures the multidimensionality of OCD in youth.

The OCI-CV is an adaptation of the adult Obsessive Compulsive Inventory – Revised (OCI-R [8]) and includes 21 items related to six symptom dimensions of pediatric OCD: (1) doubting/checking; (2) obsessing (i.e., the experience of anxiety-evoking, intrusive thoughts); (3) hoarding; (4) washing; (5) ordering; and (6) neutralization (i.e., mental strategies performed to reduce anxiety and distress, such as mental counting). Although not in complete correspondence with the major symptom dimensions obtained from the CY-BOCS checklist [9, 10], the OCI-CV dimensions capture the most common symptoms of OCD in youth and also more functional aspects of OCD such as neutralization and doubt. These features, together with its short format, makes the measure a promising tool in the assessment of pediatric OCD.

Previous validation studies of the OCI-CV, carried out in both clinical OCD samples [5, 11, 12] and community samples [12–18], have found it to be a reliable and valid measure of OCD, and to possesses a 6-factor structure. For example, in the original validation study employing a clinical sample (*N =* 100; aged 7 to 17 years) of youth with OCD, the authors observed a 6-factor structure, good test-retest reliability, and adequate sensitivity to change in the overall severity of OCD symptoms during treatment [5]. Jones et al. [11] administered the OCI-CV to youth (*N =* 96; aged 6 to 18 years) with OCD and found, after small modifications, an adequate fit of the original six-factor structure. They further found that scores on the OCI-CV dimensions, with the exception of hoarding, correlated in the moderate to large range with similar symptom dimensions assessed by the CY-BOCS. Both studies found that total scores on the OCI-CV and the CY-BOCS tended to correlated in the small range, with stronger correlations between the OCI-CV and self-report measures of depression [5, 11]. The validity of a Spanish version of the OCI-CV was carried out with a clinical sample (*N =* 94; aged 10 to 18 years) with OCD [12]. The authors observed a similar six-factor structure as in the original study, but suggested that these factors might be grouped under a second-order factor. Recently, the OCI-CV has also been proposed as an alternative measure to evaluate treatment response and clinical remission [19].

The above findings suggest that OCI-CV is a reliable and valid measure of OCD symptom dimensions in clinical samples. However, firm conclusions about the validity and clinical utility of the scale require studies employing larger clinical samples and evaluation of the OCI-CV’s psychometric properties across age groups. The latter is an important issue as OCD tends to onset early in childhood [20], and no previous study has formally compared its factor structure in younger versus older children. Finally, and with reference to the clinical utility of the OCI-CV, little is known about whether scores on OCI-CV discriminate youth with OCD from those with other mental disorders, and further evaluation is needed whether the measure is sensitive to the effects of treatment for OCD.

The primary aim of the present study was to evaluate the reliability, validity, factor structure, and clinical utility of a Swedish version of the OCI-CV in a large sample of clinically referred youth with a diagnosis of OCD. In addition to tests of its internal reliability, confirmatory factor analyses are carried out to test the applicability of the 6-factor solution, both overall and separately for younger versus older children. To further examine the validity and clinical utility of the OCI-CV, we: 1) compare scores on the OCI-CV in youth with OCD versus those with anxiety and tic disorders; 2) examine correlations between the OCI-CV, CY-BOCS, and a self-report measure of depression; and 3) evaluate whether pre- to post-treatment change scores on the OCI-CV are correlated with change scores on the CY-BOCS in youth undergoing treatment for OCD.

## Methods

### Study setting

Participants were recruited from a specialized pediatric OCD and related disorders clinic in Stockholm, Sweden, and from an outpatient child and adolescent psychiatric clinic in Lund, Sweden. At the two clinics, all patients and their parents/legal guardians routinely fill in questionnaires as a part of their initial assessments and are asked if the collected clinical data can be kept for research purposes. All participants and their caregivers in the current study provided written informed consent to the two separate, ethically-approved research projects conducted at the Stockholm (2015/1977–31/4) and Lund clinics (2015/663–3/12, 2016/92–12/5). Patients who decline participation in the research are still offered treatment or a referral to another service, according to the routine clinical procedures at each clinic.

### Participants

Participants were 563 children and adolescents aged 6 to 18 years with a current diagnosis of OCD (*n* = 434; Stockholm: *n* = 333; Lund: *n* = 101), an anxiety disorder without comorbid OCD (Lund: *n* = 84), or a Tourette’s syndrome/chronic tic disorder without comorbid OCD (Stockholm: *n* = 45). Sociodemographic and clinical characteristics of the participants are presented in Table 1. The median age was 14.20 years in the OCD group (range 6–17), 15.15 years in the anxiety disorder group (range 8–18), and 12.33 years among those with chronic tic disorders (range 7–17).

**Table 1:** Clinical characteristics and demographic variables at baseline

	OCD (*n* = 434)	Anxiety disorder (*n* = 84)	Tic disorder (*n* = 45)
Age in years, *M* (*SD*)	13.84 (2.55)	14.62 (2.57)	12.11 (2.49)
Female gender, *n* (*%*)	245 (56.45)	68 (80.95)	10 (22.22)
Parents’ highest education, *n* (%)			
Primary school	6 (1.62)	0 (0)	1 (2.86)
Secondary school	61 (16.44)	23 (32.86)	12 (34.29)
College/university > 2 years	304 (81.94)	47 (67.14)	22 (62.86)
Comorbid disorders, *n* (%)			
Any comorbid disorder	207 (47.70)	54 (69.23)	20 (44.44)
Depression	57 (13.13)	26 (30.95)	2 (4.44)
Anxiety disorder	79 (18.20)	84 (100.0)	6 (13.33)
Autism spectrum disorder	68 (15.67)	1 (1.75)	6 (13.33)
ADHD	80 (18.43)	4 (7.02)	13 (28.89)
Lifetime history of tics, *n* (%)	114 (26.33)	3 (8.57)	45 (100)
OCI-CV, *M* (*SD*)			
Total score	18.45 (7.68)	12.41 (6.20)	7.96 (5.82)
Doubting/checking	4.83 (2.73)	3.46 (2.19)	2.18 (2.06)
Obsessing	4.54 (2.20)	3.51 (1.97)	1.89 (1.60)
Hoarding	1.39 (1.67)	1.33 (1.34)	1.13 (1.20)
Washing	3.16 (2.27)	1.27 (1.73)	0.60 (1.12)
Ordering	2.92 (2.08)	2.12 (1.76)	1.71 (1.67)
Neutralizing	1.61 (1.57)	0.72 (0.97)	0.44 (0.69)

*OCD* Obsessive-Compulsive Disorder, *ADHD* Attention-Deficit/Hyperactivity Disorder, *OCI-CV* Obsessive-Compulsive Inventory – Child Version

### Measures

#### Obsessive-compulsive inventory – child version (OCI-CV [5])

The OCI-CV is a 21-item self-report measure of OCD symptoms for use with children and adolescents [5]. Each item is rated on a 3-point frequency scale (0 = *Never* to 2 = *Always*)*,* yielding a total score of 0 to 42 and six symptom dimension scores described in the Introduction. The OCI-CV was translated from English into Swedish following the recommendations of the World Health Organization for translating and adapting health-related measures [21]. The translation was made by the authors KA and PA, and was back-translated by an independent bilingual clinical psychologist. The translation was carried out after permission from the original developer (Edna Foa) who also approved the final version.

#### Children’s Yale-Brown obsessive compulsive scale (CY-BOCS [1])

The CY-BOCS is a semi-structured, clinician-rated interview of OCD symptom severity [1]. The clinician uses a checklist to identify the most frequent/disabling obsessions that are then assessed with a 5-item severity scale, with each item employing a 5-point rating (*0* = *None* to *4 = Extreme/No Control*). This process is then repeated for compulsions, and the 10 severity items are summed to yield a total severity score (range = 0 to 40), with higher scores indicating more severe symptoms. The internal consistency coefficient for the CY-BOCS in this study was Cronbach’s *α* = 0.83.

#### Children’s depression inventory – short version (CDI-S [22])

The Children’s Depression Inventory – Short version (CDI-S) is a 10-item self-report measure of depressive symptoms in children [22]. Each item is scored on a 3-point severity scale (0 to 2) with higher scores indicating more severe symptoms. The internal consistency coefficient for the CDI-S in this study was Cronbach’s *α* = 0.85.

### Procedure

At both clinics, the diagnostic status of patients was assessed using the Mini International Neuropsychiatric Interview for Children and Adolescents (MINI-KID [23]) and the CY-BOCS was administered to the OCD patients as a part of the initial clinician assessment. Participants completed the OCI-CV and CDI-S among other measures prior to treatment. For younger participants, parents were encouraged to assist their children to fill in the measures as needed. A subset of the OCD sample completed the measures again after treatment for OCD, which is part of the standard clinical procedure at the Stockholm site, and at the Lund site these follow-up assessments were carried out as part of an ongoing research on pediatric OCD. At the time of data analysis, post-treatment data was available for 83% of the participants with OCD (*n* = 359), all of which had completed a course of multimodal treatment (cognitive behavior therapy with or without concomitant medication). Of these, 77% (*n* = 277) completed the OCI-CV and CY-BOCS at both pre- and post-treatment. Data were missing at follow-up because participants failed to attend or could not be reached for a follow-up assessment, or did not complete the OCI-CV at follow-up. No differences were observed between patients with and without complete OCI-CV and CY-BOCS data for gender, age, pre-treatment depression (CDI-S) or pre-treatment OCD severity (CY-BOCS, OCI-CV).

### Statistical analyses

#### Factor structure

Confirmatory factor analyses (CFA) were undertaken to assess the fit of the six-factor solution found for the original English version of the OCI-CV to the data obtained from all participants with OCD in the present study, irrespective of their age. Three additional models, previously tested and with theoretical merit in relation to OCD heterogeneity [5, 13, 24], were also evaluated using CFAs: a second-order factor model, a single factor model, and a bi-factor model. The second-order factor model reflects a model in which covariance between the six first-order factors can be accounted for by a higher second-order factor (in contrast to the original model in which these six factors are allowed to correlate freely). The single factor model reflects a model in which there is a general OCD-factor that is related to all the symptoms assessed by the OCI-CV (i.e., a model in which no OCD heterogeneity is assumed). The bi-factor model reflects a model in which a single, general OCD factor that accounts for shared variance between all symptoms (i.e., general OCD “proneness”) is combined with the six (uncorrelated) original factors.

For the purpose of the CFAs, the individual OCI-CV items were treated as ordinal variables and robust estimations were employed. Goodness of fit measures were assessed via *χ2*, Confirmatory Fit Index (CFI), Root Mean Square Error of Approximation (RMSEA), Standardized Mean Square Residual (SRMR), and Tucker-Lewis fit Index (TLI). Adequate model fit is indicated by a lower chi-square value, CFI and TLI values > 0.95, and RMSEA and SRMR values < 0.06 [25].

To examine whether the factor structure that included six correlated first-order symptom dimensions fit the OCI-CV data obtained from younger (< 13 years; *n* = 159) and older (≥ 13 years; *n* = 275) participants with OCD, we used a multi-group CFA approach [26] in which factorial invariance was examined in a step-wise fashion. First, we tested for *configural invariance*, i.e. whether the same configural model fit the observed data in both age groups. Second, we tested for *weak invariance*, i.e. that factor loadings were similar across age groups. Third, we tested for *strong invariance*, i.e. that both factor loadings and intercepts were similar across age groups. Finally, we tested for *strict invariance*, i.e. that factor loadings, intercepts and residual variances were similar across age groups. Strict invariance was tested with theta parametrization. Factorial invariance across two models was assumed when the change in CFI (ΔCFI) was < 0.01 and the change in RMSEA (ΔRMSEA) was < 0.015 [26–28].

#### Internal consistency, convergent validity, and clinical utility

The internal consistency of the OCI-CV at baseline was calculated for the total and subscale scores using Cronbach’s *α* and coefficient omega with casewise missing data deletion. Internal consistency coefficients were estimated in the OCD group only. Acceptable internal consistency is indicated by an *α*/omega-value > 0.70 [29]. Convergent validity was investigated via zero-order Pearson correlations between total scores on the OCI-CV, the clinician-rated measure of OCD severity (CY-BOCS), and the self-report measure of depression (CDI-S).

To investigate group differences between patients with OCD, anxiety disorders, and tic disorders, an analysis of covariance (ANCOVA) was performed for the total OCI-CV score and a multivariate ANCOVA (MANCOVA) for the OCI-CV subscales. An a priori decision was made to adjust for age and gender in both models. Sidak-adjusted follow-up ANCOVAs based on the estimated marginal means were performed to examine specific group differences. Kruskal-Wallis H tests were used to examine group differences on the measures where equal variances across groups could not be assumed, as this assumption is easily violated when samples of unequal sizes are compared.

Data from the OCD participants that completed OCI-CV and CY-BOCS at pre- *and* post-treatment were used to examine sensitivity of the OCI-CV to symptom change. Specifically, to explore how well change in OCI-CV captured change in overall OCD symptoms, we estimated the zero-order Pearson correlation coefficient between baseline to post-treatment change scores on OCI-CV and CY-BOCS. We also performed paired samples t-tests for pre- to post-treatment scores on OCI-CV (total and subscale scores), CY-BOCS and CDI-S, and calculated the corresponding effect sizes (Cohen’s *d*).

#### Missing data and statistical software

There were very low rates of missing data at both clinical sites. The proportion of missing data at the item level at pre-treatment and post-treatment was 0% (both time points) at the Stockholm clinic, and 0.79 and 0.13% (respectively) at the Lund clinic. For the confirmatory factor analyses, missing data were handled by pairwise deletion. For correlation and M/ANCOVA analyses, missing data were imputed by performing an expectation-maximization algorithm before computing sum scores. The confirmatory factor analyses and estimation of coefficient omega were performed in R version 3.4.4. in R Studio version 1.1.447 using the R-packages *lavaan, psych*, and *semTools*. All other analyses were carried out in STATA v.14.2 and SPSS v.23.0.

## Results

### Factor structure

**Fig. 1:**
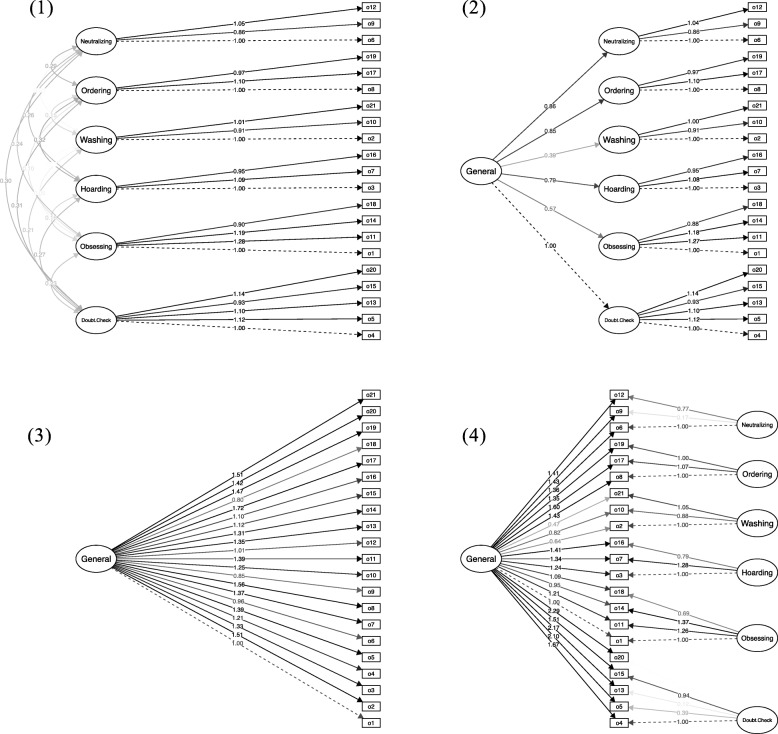
The different factor models and factor loadings. Notes. The factor models and factor loadings for (1) six correlated first-order factors, (2) six first-order factors grouped under a second-order factor, (3) a single factor, and (4) a bifactor model

Table 2 presents the model fit estimates for the four different factor models tested with CFA in the OCD sample. Figure 1 presents graphical depictions of the models as well as the factor loadings. Table 3 presents the results of the confirmatory factor analyses for the two age groups. Overall, the original six-factor model, the second-order model and the bi-factor model all provided good fits to the data, while the single factor model provided a poor fit in participants with OCD. Strict factorial invariance for the 6-factor structure was found across the two age groups, suggesting that the same 6-factor model provided an adequate fit to the data in both the younger and older groups of youth with OCD.

**Table 2:** Confirmatory factor analysis in patients with OCD

	*χ2*	*df*	CFI	TLI	RMSEA	SRMR
Original six-factor model	340.94	174	0.975	0.970	0.047	0.056
Second-order model	392.95	183	0.969	0.964	0.052	0.072
Single factor model	3166.01	189	0.561	0.512	0.192	0.205
Bi-factor model	303.67	168	0.980	0.975	0.044	0.063

*CFI* Confirmatory Fit Index, *TLI* Tucker-Lewis fit Index, *RMSEA* Root Mean Square Error of Approximation, *SRMR* Standardized Mean Square Residual

**Table 3:** Results of the multi-group confirmatory factor analysis across ages groups (< 13 years versus ≥13 years)

	df; χ2	CFI	ΔCFI	RMSEA	ΔRMSEA	SRMR
Configural	348; 489.97	0.978	–	0.044	–	0.069
Weak	363; 511.40	0.977	−0.001	0.044	0.000	0.072
Strong	378; 545.90	0.974	−0.003	0.046	0.002	0.070
Strict	399; 558.21	0.975	+ 0.001	0.043	−0.003	0.073

### Internal consistency, convergent validity, and clinical utility

The internal consistency of the OCI-CV was adequate for the total scale (*α* = 0.84; omega = 0.91), and for all but one of the subscales: doubting/checking (*α* = 0.79; omega = 0.80), obsessing (*α* = 0.77; omega = 0.81), hoarding (*α* = 0.78; omega = 0.83), washing (*α* = 0.78; omega = 0.84), and ordering (*α* = 0.86; omega = 0.85). The internal consistency coefficient for the neutralizing subscale (*α* = 0.61; omega = 0.67) was below generally agreed upon levels for acceptable internal consistency (*α*/omega > 0.70).

In respect of convergent validity, there was a small correlation between total scores on the OCI-CV and CY-BOCS at baseline (*r* = 0.19, *p* < 0.001, *n* = 429). To explore the possibility that the small correlation could be partly explained by range restriction (i.e., low variation in scores), we examined the pairwise correlation between total scores on the OCI-CV and CY-BOCS at post-treatment, where a wider range of scores were present (see Table 3). The correlation between the two measures at post-treatment was in the large range (*r* = 0.62, *p* < 0.001, *n* = 278).

The clinical utility of the OCI-CV was examined in two ways. First, and consistent with the observation that treatment-seeking youth with OCD often experience clinically significant symptoms of depression [30], a moderate correlation was observed between total scores on the OCI-CV and on a self-report measure of depression (CDI-S; *r* = 0.39, *p* < 0.001, *n* = 359). Second, we compared OCI-CV scores in participants with OCD to those with anxiety disorders and tic disorder (and no OCD), adjusting for the effects of age and gender. A significant effect for diagnostic group was found for the total score of OCI-CV (*F* (2, 558) = 41.17, *p* < .001), with participants in the OCD group scoring higher than those in the anxiety and chronic tic disorder groups (marginal means: 18.29, 8.84, and 11.89, respectively; all *p*’s < .001). The anxiety and tic disorder groups did not differ from each other (*p* = 0.171). Levene’s test of equality of error variances was not significant (*p* = .056).

A similar pattern of findings emerged for the symptom dimensions subscales of the OCI-CV in the comparison between the OCD, anxiety disorder, and tic disorder groups. After controlling for the effects of age and gender, there was a significant effect for diagnostic group on the OCI-CV subscales as a composite dependent variable (*F* (12, 1094) = 10.24, *p* < .001). For the pairwise group comparisons, Levene’s test of equality of error variances was significant (*p* < .05) for all OCI-CV subscales except for obsessing (*p* = .10). For these scales, Kruskal-Wallis H tests were also used to examine pairwise group differences. These analyses indicated that patients with OCD had significantly higher scores on all OCI-CV subscales than patients with anxiety or tic disorders, except for the hoarding subscale where no group differences emerged (OCD vs anxiety disorders, *p* = 0.93; OCD vs tic disorders, *p* = 0.94). The anxiety disorder group had higher scores on the obsessing subscale than did the tic disorder group (*p* = .03). Bonferroni corrected Kruskal-Wallis H tests directly mirrored these results. That is, significant group differences were found on all scales (all *p’s* < .001) except hoarding (*p* = .827), with higher scores in the OCD than the anxiety and tic disorder groups, and no differences between the two latter groups.

Finally, we examined the sensitivity of the OCI-CV to the effects of treatment for OCD (cognitive behavior therapy with or without concomitant medication) in participants with OCD. Table 4 presents the means and standard deviations for the pre-treatment and post-treatment scores on the OCI-CV, CY-BOCS, and CDI-S, as well as the results from the paired samples t-tests and corresponding effect sizes for all OCD participants on whom post-treatment data were available. Significant pre- to post-treatment decreases were found for the total and subscale scores of the OCI-CV, as well as for total scores on the CY-BOCS and CDI-S. Pre- to post-treatment change scores on the OCI-CV were significantly and moderately correlated with change scores over the same interval on the CY-BOCS (*r* = 0.48, *p* < .001, *n* = 277). Similarly sized correlations were found for participants recruited from the two clinical sites (Stockholm: *r* [207] = 0.40, *p* < .001; Lund: *r* [70] = 0.48, *p* < .001).

**Table 4:** Pre- and post-treatment means and standard deviations together with results for paired samples t-tests and effect sizes across study measures for the reassessed OCD participants

Measure	Pre-treatment	Min-Max	Post-treatment	Min-Max	*df*	*t*-value	*p*-value	Cohen’s *d* (95% CI)
OCI-CV								
Total score	18.17 (7.47)	3–39	11.78 (7.68)	0–33	282	13.97	< .001	0.87 (0.71, 1.03)
Doubting/checking	4.78 (2.68)	0–10	2.94 (2.39)	0–10	282	11.84	< .001	0.73 (0.57, 0.88)
Obsessing	4.55 (2.11)	0–8	3.47 (1.92)	0–8	282	9.95	< .001	0.51 (0.36, 0.66)
Hoarding	1.35 (1.59)	0–6	1.05 (1.42)	0–6	282	4.06	< .001	0.22 (0.07, 0.37)
Washing	3.06 (2.23)	0–6	1.74 (1.84)	0–6	282	11.19	< .001	0.67 (0.52, 0.83)
Ordering	2.89 (2.08)	0–6	2.04 (1.91)	0–6	282	7.70	< .001	0.43 (0.28, 0.58)
Neutralizing	1.55 (1.57)	0–6	0.89 (1.27)	0–6	282	7.62	< .001	0.49 (0.34, 0.64)
CY-BOCS	23.09 (4.61)	11–36	11.12 (6.79)	0–30	276	26.32	< .001	2.15 (1.97, 2.32)
CDI-S^a^	5.82 (3.96)	0–18	3.96 (3.94)	0–20	181	7.77	< .001	0.45 (0.27, 0.62)

*OCD* Obsessive-Compulsive Disorder, *OCI-CV* Obsessive Compulsive Inventory – Child Version, *CY-BOCS* Children’s Yale-Brown Obsessive Compulsive Scale, *CDI-S* Children’s Depression Inventory – Short Version. ^a^Data available for 182 participants

## Discussion

OCD is a highly heterogeneous and disabling condition that, if left untreated, tends to follow a chronic or recurrent course [31]. Self-report measures of OCD that can reliably capture its heterogeneity are needed as part of the overall assessment of youth with OCD [3]. The primary aim of this study was to evaluate the psychometric properties, validity and clinical utility of a Swedish translation of the OCI-CV, a self-report measure designed to capture OCD heterogeneity in children and adolescents, in a large clinical sample. A secondary aim was to extend a limited knowledge base with respect to the clinical utility of the OCI-CV by examination of its psychometric properties in younger versus older children, in youth with OCD versus other psychiatric conditions (anxiety and chronic tic disorders), and its sensitivity to the effects of treatment for OCD.

In relation to our primary aim, this Swedish translation of the OCI-CV was found to have acceptable levels of internal consistency and a factor structure similar to that reported in the English and other language versions. With respect to the latter, and consistent with the original OCI-CV validation study [5], a factor structure with six correlated, first-order factors provided a good fit to the data obtained from this large sample of clinically referred youth with a diagnosis of OCD. Additionally, acceptable fit indices were observed for models in which covariance between the symptom dimensions were accounted for by a second-order factor and by a bi-factor solution. Importantly, all of these models support the validity of the six proposed symptom dimensions, which indicates that this Swedish version of the OCI-CV can be used for multidimensional assessments of OCD in clinical samples. Furthermore, the validity of the original factor structure (i.e., six correlated first-order factors) was further supported as this structure was invariant across the younger (< 13 years) and older (≥ 13 years) children with OCD. These findings extend those of previous studies by suggesting that the major OCD symptom dimensions can be reliably assessed in both children and adolescents using the OCI-CV. When examining internal consistency, the observed results suggest that OCI-CV overall, and each of its dimensional subscales, assess the same general construct/s. Only the neutralizing subscale had low internal consistency, replicating findings from previous studies with youth [11, 16, 17] and adults [32–34]. These results may be partly explained by respondents engaging in only one or two of the three neutralizing strategies (counting, repeating, numbers) assessed by this subscale.

Consistent with two previous studies employing samples of treatment-seeking youth with OCD [5, 11], correlations between the OCI-CV and the CY-BOCS suggested low levels of convergent validity, at least as assessed at pre-treatment. This finding may be partly explained by *common method bias*, that is, measures of the same modality (e.g., self-report) tend to show stronger associations than measures of different modalities (e.g., self-report and interview [35]). Consistent with this explanation and findings from previous studies, a much stronger association was found between the self-report measures of OCD (OCI-CV) and depression (CDI-S), than between the OCI-CV and CY-BOCS [5, 11], which is not surprising given the positive association between OCD and depressive symptoms [30]. Another possible explanation for the small pre-treatment correlation between OCI-CV and CY-BOCS is the difference in the scoring of the two scales. Unlike the OCI-CV, the CY-BOCS is designed such that the total symptom severity score is independent of the number and type of obsessions and compulsions. Thus, a patient with severe symptoms within only one OCD dimension would receive a low total score on the OCI-CV but could have a high total score on the CY-BOCS because these few symptoms were rated as frequent and highly impairing by the interviewer. A third and non-incompatible possibility, suggested by the strong correlation between the OCI-CV and CY-BOCS at post-treatment, is that the small correlation between the two measures at pre-treatment reflects low levels of variability in CY-BOCS scores in clinically referred youth who have not yet begun treatment for this condition [36].

In relation to our secondary aim, the present results suggest that the OCI-CV is a sensitive and clinically useful measure as part of an overall assessment of OCD symptoms in children and adolescents. First, when compared to clinically referred youth with anxiety and chronic tic disorders, participants with OCD scored significantly higher on both the total score and subscale scores of OCI-CV. This suggests that scores on OCI-CV are more specific to the severity of OCD than to the symptoms of two related conditions (anxiety and chronic tic disorders). The one exception to this finding was the hoarding subscale, where the three diagnostic groups did not differ. This finding could be due to a floor effect as all three groups had low scores on the hoarding subscale. Furthermore, converging research suggests that hoarding symptoms are not uniquely or specifically associated with OCD but, rather, are equally common in other emotional disorders [37, 38], hence the separate status of hoarding disorder in the 5th edition of the Diagnostic and Statistical Manual of Mental Disorders (DSM-5 [39]) and the 11th edition of the International Classification of Diseases (ICD-11 [21]).

As stated above, the clinician-administered CY-BOCS is the recommended and most widely-used measure for assessing outcomes in OCD treatment trials. Participants who received multi-modal treatment (cognitive behavior therapy with or without concomitant medication) for OCD experienced significant pre- to post-treatment reductions in total OCD severity with a large effect size as measured by the CY-BOCS. Likewise, significant pre- to post-treatment decreases were observed for the total and subscale scores of the OCI-CV. The effect sizes varied between the total and the different subscale scores, with the largest being for the full scale and lowest for the hoarding subscale. Further, change scores on the OCI-CV and the CY-BOCS were significantly correlated in the moderate range. These findings suggest that OCI-CV captures change in overall OCD severity reasonably well, and may be used alongside the CY-BOCS to provide complementary information about improvement in specific symptom dimensions, which is in line with the findings and recommendations by McGuire et al. [19] for assessing treatment response based on the OCI-CV.

The present study benefited from several methodological strengths including a large sample of treatment-seeking youth with OCD, anxiety, and tic disorders, all of whom were assessed with structured diagnostic interviews, and many of whom provided post-treatment data. However, the present results need to be viewed within the context of certain methodological limitations. First, our findings are based on youth referred to two clinics in Sweden, and a non-clinical comparison group was not included. Additional investigations in a Swedish context are needed to further establish the validity and clinical utility of the OCI-CV, including evaluation of clinical cut-off scores to be used in Swedish settings. Second, the clinician-rated CY-BOCS was chosen to evaluate the convergent validity of the OCI-CV for pragmatic reasons, i.e. the CY-BOCS is part of the routine assessment of youth with OCD in the two clinics where participants were recruited. Further investigations are needed of the convergent validity of the OCI-CV relative to other measures of OCD, particularly those assessing the major OCD symptom dimensions, and relative to measures of constructs that may help explain variation in the severity of these dimensions, e.g. the Obsessive Belief Questionnaire-Child Version (OBQ-CV [40]), the Obsessive-Compulsive Trait Core Dimensions Questionnaire (OCTCDQ [41–43]), and the Disgust Emotion Scale – Child Version (DES-C [42–44]), would be of interest. Finally, limited information was available on the length and content of treatment in the OCD group, and in the absence of a no-treatment control, our findings for the sensitivity of the OCI-CV to the effects of OCD treatment should be considered preliminary.

## Conclusions

The Swedish version of the OCI-CV is a brief and easily administered self-report measure of OCD and its symptom dimensions. It possesses good psychometric properties and appears to be a valid and clinically useful measure of pediatric OCD, used alongside the CY-BOCS in clinical settings as a measure of OCD in youth.

## Data Availability

The datasets generated and analysed during the current study are not publicly available due to national (Swedish) and EU legislation, but are available from the corresponding author on reasonable request.

## Abbreviations

ANOVA: Analysis of covariance
CDI-S: Children’s Depression Inventory – Short version
CFA: Confirmatory factor analyses
CFI: Confirmatory Fit Idex
C-FOCI: Children’s Florida Obsessive Compulsive Inventory
ChOCI: Children’s Obsessional Compulsive Inventory
CY-BOCS: Children’s Yale-Brown Obsessive Compulsive Scale
DES-C: Disgust Emotion Scale – Child Version
DSM-5: 5th edition of the Diagnostic and Statistical Manual of Mental Disorders
ICD-11: 11th edition of the International Classification of Diseases
MANOVA: Multivariate analysis of covariance
MINI-KID: Mini International Neuropsychiatric Interview for Children and Adolescents
OBQ-CV: Obsessive Belief Questionnaire-Child Version
OCD: Obsessive-compulsive disorder
OCI-CV: Obsessive Compulsive Inventory – Child Version
OCI-R: Obsessive Compulsive Inventory – Revised
OCTCDQ: Obsessive-Compulsive Trait Core Dimensions Questionnaire
RMSEA: Root Mean Square Error of Approximation
SRMR: Standardized Mean Square Residual
TLI: Tucker-Lewis fit Index
